# Demonstration of *Allium sativum* Extract Inhibitory Effect on Biodeteriogenic Microbial Strain Growth, Biofilm Development, and Enzymatic and Organic Acid Production

**DOI:** 10.3390/molecules26237195

**Published:** 2021-11-27

**Authors:** Viorica Maria Corbu, Irina Gheorghe, Ioana Cristina Marinaș, Elisabeta Irina Geană, Maria Iasmina Moza, Ortansa Csutak, Mariana Carmen Chifiriuc

**Affiliations:** 1 Department of Genetics, Faculty of Biology, University of Bucharest, Botanical Garden, 3 Intrarea Portocalelor St., 050095 Bucharest, Romania; viorica.corbu@yahoo.com (V.M.C.); cs.ortansa@gmail.com (O.C.); 2 Research Institute of the University of Bucharest—ICUB, 91-95 Splaiul Independenței St., District 5, 050095 Bucharest, Romania; iasmina_moza@yahoo.com (M.I.M.); carmen.chifiriuc@gmail.com (M.C.C.); 3 Doctoral School of Biology, University of Bucharest, 91-95 Splaiul Independenței St., District 5, 050095 Bucharest, Romania; 4 Department of Microbiology and Immunology, Faculty of Biology, University of Bucharest, Botanical Garden, 3 Intrarea Portocalelor St., District 6, 060101 Bucharest, Romania; 5 National R&D Institute for Cryogenics and Isotopic Technologies—ICIT, Rm. Valcea, 4 Uzinei St., 240050 Ramnicu Valcea, Romania; irina.geana@icsi.ro; 6 Romanian Academy of Scientists, 54 Spl. Independentei St., District 5, 50085 Bucharest, Romania; 7 The Romanian Academy, 25, Calea Victoriei, Sector 1, District 1, 010071 Bucharest, Romania

**Keywords:** filamentous fungi, garlic extract, biodeterioration, biocides, antifungal

## Abstract

To the best of our knowledge, this is the first study demonstrating the efficiency of *Allium sativum* hydro-alcoholic extract (ASE) againstFigure growth, biofilm development, and soluble factor production of more than 200 biodeteriogenic microbial strains isolated from cultural heritage objects and buildings. The plant extract composition and antioxidant activities were determined spectrophotometrically and by HPLC–MS. The bioevaluation consisted of the qualitative (adapted diffusion method) and the quantitative evaluation of the inhibitory effect on planktonic growth (microdilution method), biofilm formation (violet crystal microtiter method), and production of microbial enzymes and organic acids. The garlic extract efficiency was correlated with microbial strain taxonomy and isolation source (the fungal strains isolated from paintings and paper and bacteria from wood, paper, and textiles were the most susceptible). The garlic extract contained thiosulfinate (307.66 ± 0.043 µM/g), flavonoids (64.33 ± 7.69 µg QE/g), and polyphenols (0.95 ± 0.011 mg GAE/g) as major compounds and demonstrated the highest efficiency against the *Aspergillus versicolor* (MIC 3.12–6.25 mg/mL)*, A. ochraceus* (MIC: 3.12 mg/mL)*, Penicillium expansum* (MIC 6.25–12.5 mg/mL), and *A. niger* (MIC 3.12–50 mg/mL) strains. The extract inhibited the adherence capacity (IIBG% 95.08–44.62%) and the production of cellulase, organic acids, and esterase. This eco-friendly solution shows promising potential for the conservation and safeguarding of tangible cultural heritage, successfully combating the biodeteriogenic microorganisms without undesirable side effects for the natural ecosystems.

## 1. Introduction

The biodeterioration of tangible cultural heritage is mainly due to fungi and bacteria, harboring a huge reservoir of microbial enzymes (e.g., cellulases, hemicellulases, endoglucanases, cellobiohydrolases, esterases, phenoloxidases, pectinases, amylases) and acids (gluconic, citric, and oxalic). Moreover, the pigment production as well as biofilm formation cause aesthetic and structural alteration or loss of cultural heritage objects and buildings [1]. Deterioration caused by biological agents has been reported for different classes of materials: wool, paintings, textiles, paper, parchment, leather, etc. [1,2,3].

In Romania, only several studies have focused on the biodeterioration of material cultural heritage and on the diversity of the involved genera and species. These studies have highlighted the presence of different filamentous fungi, such as *Alternaria* spp., *Penicillium* spp. (*P. rugulosum, P. digitatum, P. brevicompactum*, and *P. chrysogenum*), *Aspergillus* spp., *Rhizopus* spp. (*R. stolonifer*), *Fusarium* spp. *(F. proliferatum*), *Mucor* spp. (*M. circinelloides*), *Trichoderma* spp. (*T. longibrachyatum*), *Botrytis* spp., *Planomicrobium* spp., *Cladosporium* spp., *Variovorax* spp., and *Candida* spp. on wooden walls, stone walls, and mural paintings of heritage churches from different counties, as well as on different ethnographical textiles, part of the collection of The National Museum of the Romanian Peasant [1,4,5,6,7,8,9,10].

In this context, there is an increased necessity to develop effective and ecofriendly prevention and conservation solutions to protect the material cultural heritage against microbial (re)colonization, with low toxicity risk for human, animal, and environmental health [11,12]. Our research group revealed the efficiency of MgB_2_ ecofriendly powders against biodeteriogenic fungi isolated from wooden and stone churches and heritage objects from Romanian counties [13].

One of the valuable ecological alternatives to common synthetic antifungal agents with a relatively safe and widely accepted status, due to the low risk of side effects [11] and minimal ecotoxicity being represented by the use of plant extracts, knowing their previously demonstrated antimicrobial and antibiofilm activity against fungal and bacterial strains, causing the deterioration of cultural heritage [14,15]. Until the present, in Romania, there are very few studies regarding the antimicrobial activity of different plant extracts against biodeteriogenic microorganisms of cultural heritage objects and buildings [16,17]. Taking into account the fact that for more than 5000 years, garlic has been recognized as a medicinal plant [18], with its antimicrobial activity mainly due to the allicin [19], this research proposes an ecofriendly solution against biodeteriogenic microorganisms on the basis of an *Allium sativum* extract [20].

In this context, we aimed to evaluate the capacity of the hydro-alcoholic (50:50, %) extract of *A. sativum* to inhibit the biodeteriogenic fungi and bacteria grown in planktonic and biofilm states and to examine the effect of garlic bulb extract on the production of soluble enzymes and organic acids. The physico-chemical characterization of *A. sativum* extract was first performed, followed by the in vitro demonstration of the efficacy of this alternative solution on a collection of over 200 filamentous fungi and bacterial strains isolated from wooden and stone biodeteriorated heritage objects and churches from different Romanian counties.

## 2. Results

### 2.1. Chemical Composition and Antioxidant Activity

The results obtained for the total polyphenol content and antioxidant activity by different methods are shown in Table 1. The results of the antioxidant activity of the extracts obtained by different assays are slightly different and may be due to differences in the used methods [21]. It can be noticed that the *A. sativum* extract had a more pronounced reducing capacity, revealed by the DPPH method, as compared to the CUPRAC and FRAP assays. The total thiosulfinate content obtained for our *A. sativum* fresh plant material was significantly higher than that reported by Gonzalez et al. (2009) (2.65 to 4.59 mM/100 g) [22], probably due to lower processing of plant material.

**Figure 1:**
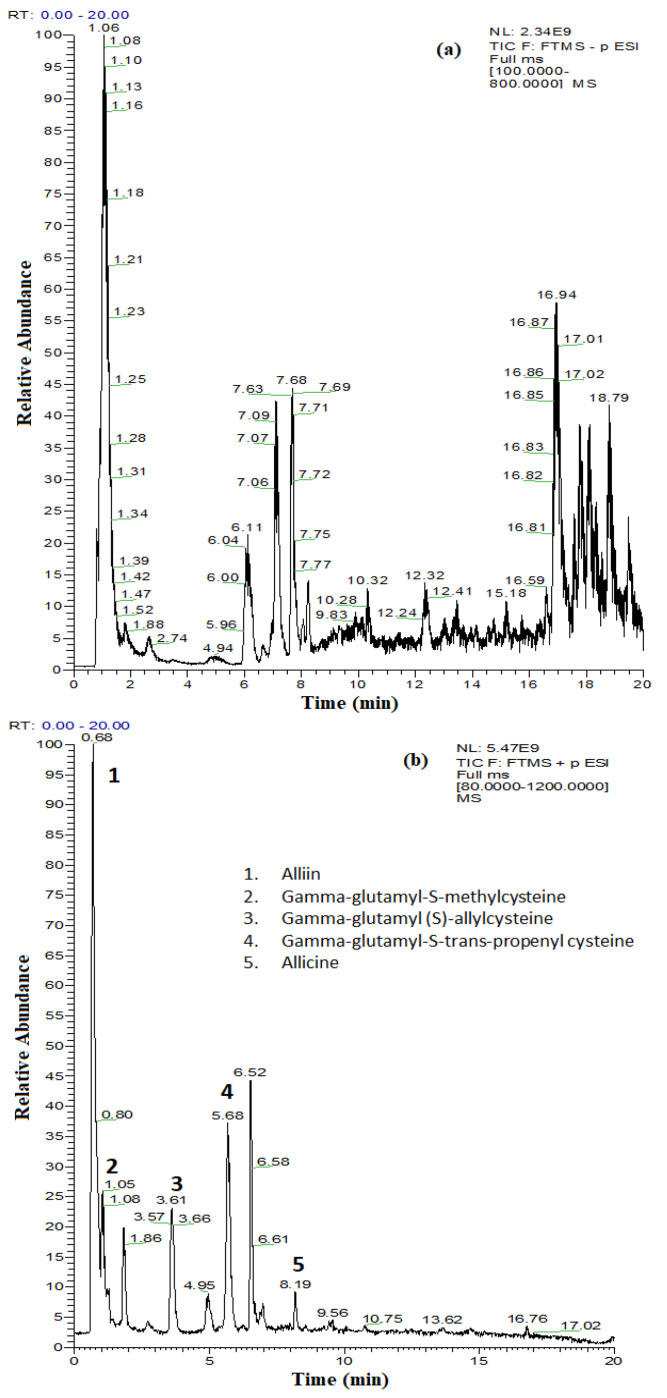
The obtained total ion current (TIC) chromatograms for the separation of (**a**) polyphenolic compounds from *A. sativum* plant extract by UHPLC–MS/MS detection in negative ionization mode and (**b**) thiosulfinate compounds from *A. sativum* plant extract by UHPLC–MS/MS detection in positive ionization mode.

The obtained chromatograms from the UHPLC–MS/MS analysis of *A. sativum* plant extract are shown in Figure 1a for polyphenolic compounds and Figure 1b for thiosulfinate compounds.

Most of the phenolic compounds in the *A. sativum* plant extract were detected at trace levels, with concentrations below 10 µg/L, while the contents of vanillic, t-ferulic, and ellagic acids and epicatechin were 65.2 µg/L, 18.8 µg/L, 13.2 µg/L, and 41.2 µg/L, respectively (Table 2). Even if the polyphenol content of the *A. sativum* plant extract is low, the total of the quantified phenolic acids (128 µg/L) and of the quantified flavonoids (97.2 µg/L) guarantees the inhibitory activity on biodeteriogenic fungi spore germination and mycelium growth through the synergistic effect of the antimicrobial compounds [23,24].

In the absence of standards, identification of other compounds in the *A. sativum* plant extract was based on the search for the protonated molecules [M − H]^+^ and comparisons with the specific literature. The exact mass search using ChemSpider reference library enabled us to identify thiosulfinated compounds with antimicrobial activity (alliin, gamma-glutamyl-S-methyl cysteine, gamma-glutamyl-(S)-allyl cysteine, gamma-glutamyl-S-trans-propenyl cysteine, and allicin) (Figure 1b, Table 2). The reactivity of thiosulfinates to the thiol groups is an important component of the antimicrobial activity. In the case of allicin, in addition to being redox-active, it is quite lipophilic, allowing for the permeability of cell membranes. It has been demonstrated that allicin is able to form transient pores in artificial membranes and bio-membranes [25,26,27]. The identification of allicin in the *A. sativum* plant extract (TIC, extracted chromatogram and the extracted ion chromatogram for *m*/*z* 163.0245) is presented in Figure S1.

Because the content of phenolic compounds is low, we can assume that the antioxidant activity is due to organosulfur compounds.

### 2.2. Qualitative Screening of the Antimicrobial Activity

#### 2.2.1. Antifungal Activity

**Figure 2:**
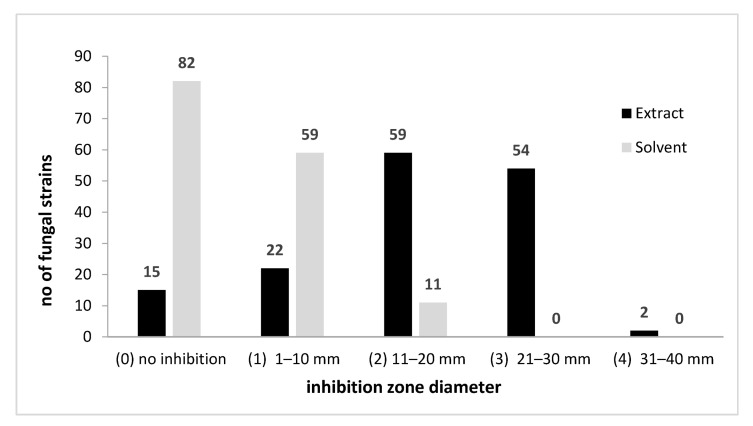
*A. sativum* inhibitory activity against fungal strains expressed by arbitrary units of growth inhibition zone diameter.

**Figure 3:**
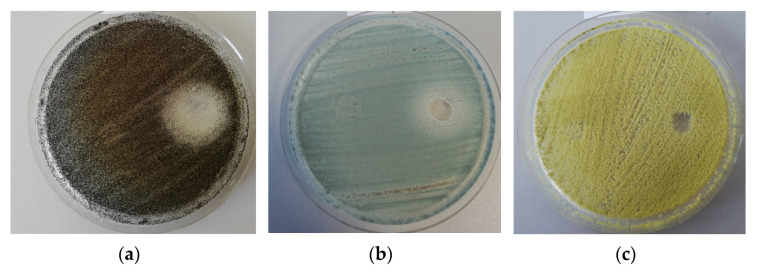
Example of the disk diffusion screening assay of the antimicrobial activity of the tested *A. sativum* extract against *A. niger* (**a**), *P. chrysogenum* (**b**), and *A. flavus* (**c**) strains isolated from the wooden churches (the obverse of the colonies).

A total number of 152 filamentous fungi strains previously isolated from wooden and stone churches (narthex, nave, altar) as well as from museum objects (paper, textiles, and paintings) and characterized by MALDI-TOF mass spectrometry were used in this study. These strains belong to eight genera with 19 species (*Penicillium chrysogenum*, *P. corylophylum*, *P. expansum*, *P. digitatum*, *Aspergillus niger*, *A. flavus*, *A. clavatus*, *A. nidulans*, *A. ochraceus*, *A. versicolor*, *A. ustus*, *A. sydowi*, *Rhizopus oryzae*, *Trichoderma orientale*, *Byssochlamys spectabilis*, *Alternaria alternata*, *Purpureocillium lilacinum*, *Fusarium proliferatum*, *F. cerealis culmorum group*, *Cladosporium* spp.). The strains belonging to the *Penicillium* and *Aspergillus* genera were the most frequently isolated from the heritage objects and churches. The qualitative (adapted disc diffusion) method was used for the screening of the antimicrobial activity of the *A. sativum* plant extract. The antimicrobial activity of the tested plant extract against the tested microfungi demonstrated a very high efficiency against most of the tested strains (Figure 2 and Figure 3 and Figure S2). The garlic extract was more efficient than the used solvent in terms of inhibitory effect, with more than 100 tested fungal strains responding to the extract treatment with the occurrence of a growth inhibition zones larger than 10 mm as compared to only 11 strains in case of the solvent (Figure 2).

#### 2.2.2. Antibacterial Activity

**Figure 4:**
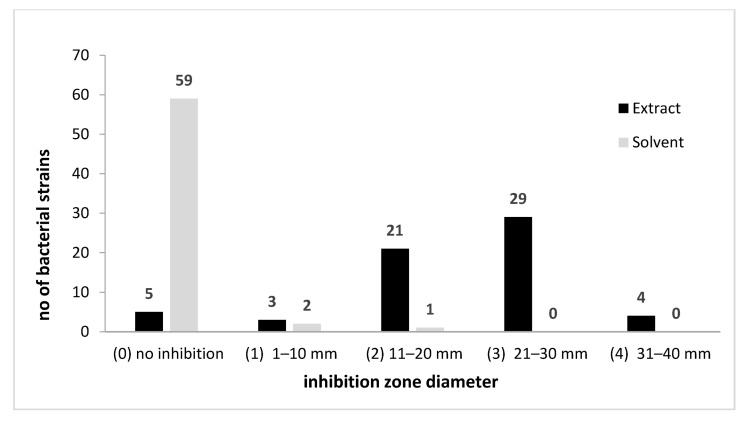
*A. sativum* inhibitory activity against bacterial strains expressed by arbitrary units of growth inhibition zone diameter.

**Figure 5:**
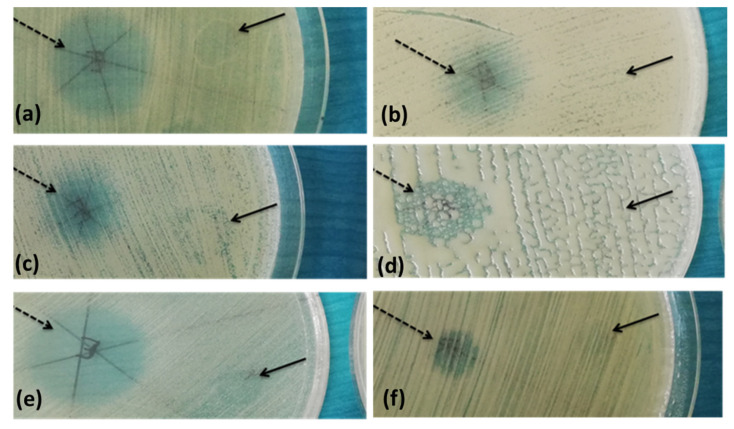
Example of the disk diffusion screening assays of the antimicrobial activity of the tested *A. sativum* extract against *B. cereus* (**a**), *B. pumilus* (**b**,**c**), *B. megaterium* (**d**), *R. erythrophyllus* (**e**), *A. globiformis* (**f**) strains; dashed arrow—*A. sativum* inhibition zone, full arrow—solvent inhibition zone.

The qualitative screening of the antimicrobial activity of the *A. sativum* plant extract was also performed against 62 bacterial strains previously isolated from biodeteriorated wooden, stone, textile, or paper objects. These strains belong to four genera and 10 species (*Bacillus pumilus*, *B. megaterium*, *B. subtilis*, *B. cereus*, *B. atrophaeus*, *B. thuringiensis*, *Pseudomonas koreensis*, *R. erythrophyllus*, *Arthrobacter globiformis*, *A. aurascens*). The garlic extract revealed a very high efficiency on most bacterial strains; the inhibitory effect, with only few exceptions (eight strains mainly isolated from the external surfaces of the wooden churches), was due to the extract and not to the solvent (Figure 4 and Figure 5 and Figure S2).

### 2.3. Quantitative Evaluation of the Antimicrobial Activity

#### 2.3.1. Antifungal Activity

**Figure 6:**
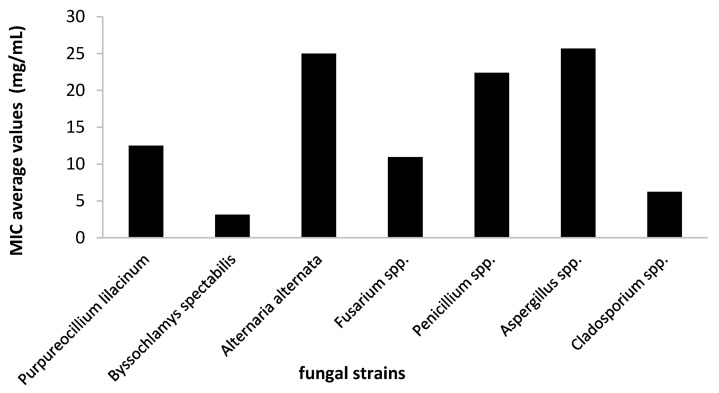
Average values of minimal inhibitory concentration (MIC) of *A. sativum* extract obtained on the tested fungi.

For this step, the plant extract was tested for its antifungal activity against the most susceptible strains, as evidenced in the screening assay. Therefore, the efficiency of *A. sativum* hydro-alcoholic extract was expressed by the MIC values that were determined using the binary serial dilution method. The strains that responded better to the treatment were identified as *B. spectabilis*, *Cladosporium* spp., and *T. orientale*, and at the opposite side, the strains belonging to the *Alternaria*, *Penicillium* and *Aspergillus* genera were less susceptible, with MIC approximately two times higher than the average values (Figure 6).

**Figure 7:**
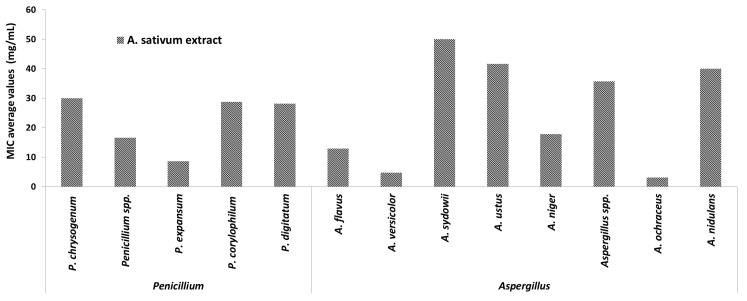
Average values of minimal inhibitory concentration (MIC) of *A. sativum* extract on strains belonging to *Penicillium* and *Aspergillus* genera.

Regarding the MIC distribution for different species of the two most frequently encountered genera involved in the biodeterioration of the cultural heritage churches and objects (*Penicillium* spp. and *Aspergillus* spp.) (Figure 7), it was shown that *A. sativum* plant extract demonstrated the highest efficiency against *A. ochraceus* (MIC = 3.12 mg/mL) and *A. versicolor* strains (MIC = 4.16 mg/mL), followed by the *P. expansum* and *A. niger* strains.

**Figure 8:**
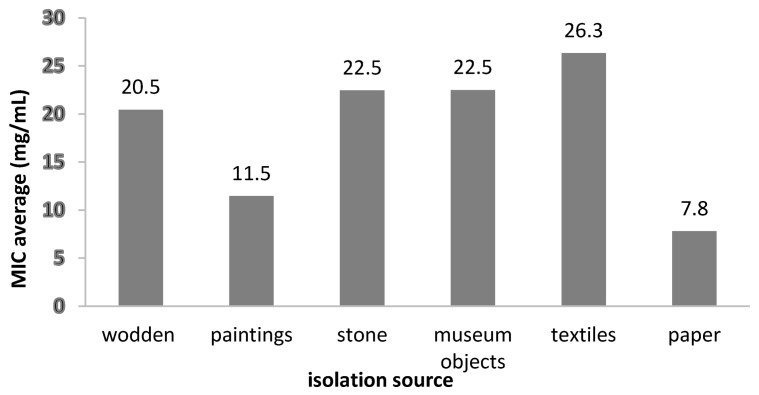
The average MIC values of the *A. sativum* extract on fungal strains, depending on the isolation sources.

The MIC value distribution by isolation sources demonstrated that the most susceptible strains were from the biodeteriorated paper (MIC = 7.81 mg/mL), while the least susceptible were from textiles and stone materials (MIC = 26.33 mg/mL and 22.5 mg/mL) (Figure 8).

The activity of *A. sativum* extract against *Penicillium* spp. was significantly higher in comparison with that of the solvent activity for strains isolated from different sources: wooden (*p* < 0.00001), painting (*p* < 0.05), stone (*p* < 0.05), and textiles (*p* < 0.05). In the case of *Aspergillus* spp., the antimicrobial activity of *A. sativum* extract was significantly higher than that of the solvent for wooden (*p* < 0.001) and textile (*p* < 0.05) strains. Moreover, statistically significant MIC values of the extract compared to the solvent control were obtained for wood strains belonging to *Fusarium* (*p* < 0.05) and *Cladosporium* (*p* < 0.05) genera.

#### 2.3.2. Antibacterial Activity

**Figure 9:**
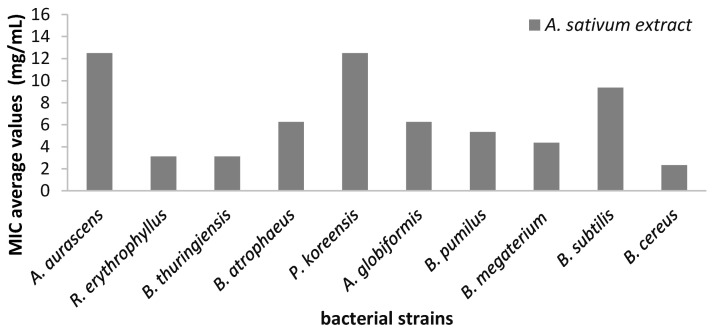
Average values of minimal inhibitory concentration (MIC) of *A. sativum* extract on different bacterial strains.

The quantitative evaluation of the antimicrobial efficiency of *A. sativum* hydro-alcoholic extract against bacterial strains revealed the highest efficiency against *R. erythrophyllus* and *B. thuringiensis* strains (MIC = 3.12 mg/mL) (Figure 9).

**Figure 10:**
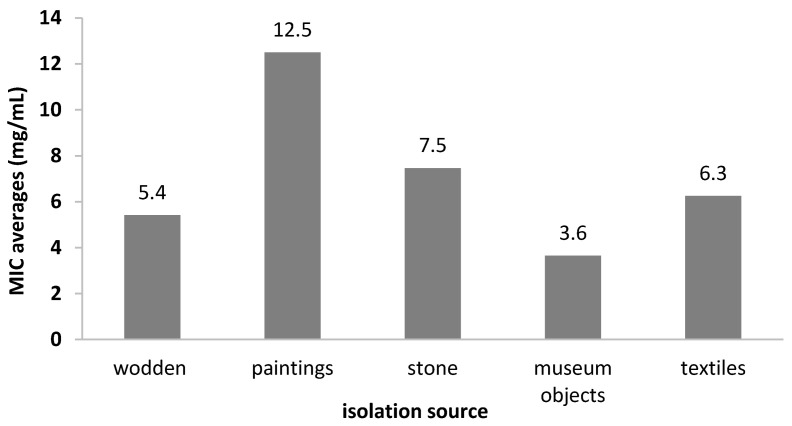
The average MIC values of the *A. sativum* extract depending on bacterial strain isolation sources.

Regarding the MIC values distribution by the isolation source, we showed that the most susceptible strains were the isolates from the museum collections (MIC = 3.64 mg/mL), while those from mural paintings were the least susceptible (MIC = 12.5 mg/mL) (Figure 10).

In the case of bacterial strains, a statistically significant difference was observed between the activity of the extract and solvent for *Bacillus* spp. strains isolated from wood (*p* < 0.01) and stone (*p* < 0.01). In addition, for the *Purpureocillum* spp. strains isolated only from stone, the difference between the solvent control and extract was also significant (*p* < 0.05).

### 2.4. Antibiofilm Activity

In Table 3, the fungal strains isolated from textiles showed the ability to adhere to the inert surface. Among the isolated fungi, it was observed that 2 out of the tested 17 strains did not show the ability to adhere to the inert substratum. In the case of adherent strains, the extract inhibited the microbial adhesion, with the inhibition index of biofilm growth (IIBG) varying from 80.47 ± 2.73 to 95.08 ± 0.91. In the case of bacterial strains, from the 19 tested strains, only three had the capacity to form microbial biofilms, all being inhibited with an IIBG of 44.62 ± 7.30 to 87.56 ± 4.60. Apart from two strains (*P. chrysogenum* from wood and *B. arthopahaeus* from stone), in all cases, the extract was proven to be more efficient than the solvent, with the results being statistically significant.

### 2.5. Effects of A. sativum Extract on Microbial Soluble Factor Production

Among the tested fungal strains, 34.21% produced cellulases, 52.63% produced acids, and 34.21% produced esterase. No biodeteriogenic strain produced phenoloxidases.

The *A. sativum* extract inhibited or stimulated the production of biodeteriorating factors (cellulase, phenoloxidase, esterase, and organic acids), with the effect depending on the tested microbial strain. *A. sativum* extract inhibited cellulase production in the case of 61.54%, decreased acidity for 55%, and inhibited esterase production for 23.08% from the total of tested fungal strains. Cellulase inhibition was identified, especially for strains isolated from stone and textile, while acidity was reduced for strains isolated from wood and textile.

Table 4 shows only the variants for which differences were observed compared to the untreated strain control. The *A. sativum* extract significantly inhibited the cellulase production in the case of four fungal strains (*Aspergillus* spp., *Penicilium* spp., and two *P. corylophilum*), the esterase production in one bacterial (*B. subtilis*) and three fungal strains (two *Aspergillus* spp., one *A. flavus*), and the organic acid production in the case of seven fungal (two *A. niger,* two *P. chrysogenum*, one *A. sydowii, P. lilacium*, and *Aspergillus* spp.) and two bacterial strains (*B. megaterium* and *B. cereus*) (*p* < 0.05).

## 3. Discussion

This study was conducted to reveal the efficiency of *Allium sativum* hydro-alcoholic extract as an eco-friendly solution to combat fungi and bacteria involved in biodeterioration of cultural heritage buildings and objects. The bioevaluation of the proposed solution was performed on a significant collection of more than 200 biodeteriogenic microbial strains previously isolated from churches and objects from different Romanian counties and characterized by culture dependent and independent methods.

We demonstrated that the antimicrobial activity of *A. sativum* hydro-alcoholic extract against biodeteriogenic fungal and bacterial strains is associated with the presence of allicin and thiosulfinic compounds identified by UHPLC that, from over 100 biologically active compounds derived from garlic, are known for their broad spectrum of antifungal activity [20,28]. However, the antimicrobial activity was tested in most studies against plant, animal, and human pathogenic strains. Choo et al., in 2020 showed that allicin could serve as a potential alternative strategy effective against *A. fumigatus*, *A. niger*, *A. versicolor*, *A. terreus* and *Candida* spp. responsible for human infections in immunocompromised patients [29]. Allicin acts on the cell wall, causing pore formation and thus promoting the action of synthetic fungicides, such as flucytosine and amphotericin B [30]. Actually, allicin can disrupt the cell’s electrochemical potential and thus induce apoptosis in yeasts [31]. In general, disintegration of cytoplasm, breakdown of the cell membrane and cell wall, and collapse of hyphae were observed when fungi were treated with allicin and garlic extract [32]. Other authors have revealed that allicin could alter RNA and lipid or acetyl-CoA formation [33,34]. It has been demonstrated that allicin exhibited antifungal activities in vitro and in vivo against common plant pathogenic fungi [35,36]. Allicin has been shown to exhibit broad-spectrum antimicrobial activity against multidrug-resistant Gram-positive bacteria (e.g., *S. aureus* strains) [37]. Other major chemical compounds identified in this study are reported as having antimicrobial activity although very few of them have been tested against biodeteriogenic fungi. A study conducted by [38] showed that stilbenoids (resveratrol and some of its derivatives) are very effective against phytopathogenic fungi such as *A. flavus, P. italicum, A. terreus*, and *F. verticillioides* and recommends them as food preservatives. Similarly, Oliveira et al., in 2015, proved that quercetin and rutin might act as enhancers of amphotericin B antifungal activity and reduced its cytotoxic effect against red blood cells [39]. Rutin acts as a pro-oxidant that induces ROS generation and subsequently causes cell membrane disruption and protein denaturation [40]. p-Coumaric acid can inhibit *F. oxysporum* and *F. verticillioides* at low MIC values (below 576 µg/mL) but has no effect against *P. brevicompactum*, *P. expansum*, *A. flavus*, and *A. fumigatus*, which are frequently isolated from heritage buildings. Regarding the antibacterial activity of p-coumaric acid and its derivatives (caffeic acid, ferulic acid, and chlorogenic acid), a significant inhibitory potential was recorded at concentrations higher than 1 mg/mL when tested against bacterial strains such as *S. aureus, S. epidermidis, S. agalactiae*, or *E. coli* [41]. Although limited potential was described until now, some of the compounds from our garlic extract such as vanillin and p-coumaric acid might act as mycotoxin inhibitors [42], which is why such a research direction of should be addressed, taking into account the fact that many of the biodeteriogenic fungi can produce mycotoxins.

*A. sativum* extract can represent an eco-friendly solution to combat biodeteriogenic strains belonging to bacteria (*Arthrobacter* spp., *Bacillus* spp., *Micrococcus* spp., *Paracoccus* spp.) or fungi (*Alternaria* spp., *Aspergillus* spp., *Penicillium* spp., *Phoma* spp., *Fusarium* spp., *Cladosporium* spp.) recovered from a stonework surface [43]; Kutawa et al., in 2018, showed that ethanolic *A. sativum* extract is active on *Fusarium* spp. and *Rhizopus* spp. (2.5 mg/mL and 5.0 mg/mL for *Fusarium* spp. and *Rhizopus* spp., respectively) [44]. Yetgin et al., in 2018, demonstrated that the antimicrobial activities of two samples of *A. sativum* alcoholic extracts from Turkey and China against seven genera of bacterial strains and *C. albicans* depended on the geographic origin of the plant [14].

The antimicrobial activity of *A. sativum* extract was also evaluated against *B. subtilis*, *Micrococcus luteus*, *P. chrysogenum*, and *Aspergillus* spp. strains isolated from colonized artworks in Italy, suggesting the possible use in the control of biodeterioration of cultural heritage, safe both for human health and the environment [45]. In Romania, until the present, the only antimicrobial activity that has been demonstrated is of the *Ocimum basilicum* and *A. ursinum* hydro-alcoholic extracts and essential oils against *Aspergillus* spp., *Penicillium* spp., and *Mucor* spp. recovered from paper artefacts [16,17].

Biofilm formation is one of the first steps involved in biodeterioration of historic monuments followed by secretion of different enzymes that affect the substrate [46], which is why the development of strategies to prevent the adhesion of biodeteriogenic microorganisms to the surface of the heritage objects is highly necessary. This study demonstrated that the most adherent biodeteriogenic fungal strains were isolated from textiles, followed by wooden and stone objects, and a significant inhibition of microbial adhesion was obtained in the presence of garlic extract. Many of the chemical compounds identified in the garlic extract, although not directly tested on biodeteriogenic microbial strains, have been described as having anti-adhesion activity. A study conducted by Deepika et al. in 2016 shows that rutin inhibits biofilm formation by *P. aeruginosa*, and its effect might be improved by combining it with small doses of gentamicin [47]. Moreover, gallic acid showed good inhibitory action both on growth and biofilm formation in the case of Gram-negative bacteria, Gram-positive bacteria [48], and *C. albicans* [49]. Similarly, p-coumaric acid and gallic acid are involved in decreasing the rate of *P. fluorescence* colonization on abiotic surfaces by reducing the expression level of *flgA* gene, which is responsible for flagella production [50]. Allicin is another compound that can exert anti-adhesion activity against a wide variety of microorganisms, including filamentous fungal species such as *A. flavus, A. fumigatus, A. niger, A. terreus*, and *A. versicolor* when administered in combination with various fungicides such as amphotericin B [29]. Allicin might also inhibit polysaccharide intercellular adhesins synthesis, which is required for the biofilm formation in *S. epidermidis* [51]. Moreover, it reduces exopolysaccharide production in *P. aeruginosa* and downregulates the expression of different virulence factors involved in quorum sensing, an intercellular communication mechanism involved in the regulation of biofilm development [52]. A study conducted by Girish et al. in 2019 presents a polymeric matrix functionalized with garlic extract as a strategy with high efficiency in penetrating and disrupting the methicillin-resistant *S. aureus* biofilms [53].

Deteriogenic fungal strains produced cellulase, esterase (34.21% of each enzyme type), and organic acid (52.63%). A reduction or inhibition of cellulose/organic acid/esterase production by 61.54%/55%/23.08% was observed for the analyzed fungal strains grown in the presence of garlic extract. The inhibitory action of *A. sativum* extract against enzyme production can be attributed to the presence of alliin, gamma-glutamyl-S-methyl cysteine, gamma-glutamyl-(S)-allyl cysteine, gamma-glutamyl-S-trans-propenyl cysteine, and allicin as the major antifungal components.

Microbial strains, due to their metabolic diversity, designed to ensure their survival in different environments, are the main responsible sources of heritage object deterioration. For the stone monuments, microorganisms are responsible for pigment production and structural damage due to their ability to penetrate the depth of the support. Moreover, some of them are able to produce organic and inorganic acids and to determine salt crystallization that, in the end, causes discoloration and erosion [54]. For wood and paper biodeterioration, lignocellulolytic microorganisms play the main role [55,56], while in the case of man-made textiles, esterase-producing microbial strains can cause significant damage. Similarly, the deterioration of paintings is caused by a multitude of enzymes produced by biodeteriogenic microorganisms such as amylase, lipase/esterase, protease, and acids [52,55,56]. A study conducted by El Hassni et al. in 2021 showed that caffeic acid, p-coumaric acid, and ferulic acid have great inhibitory action on hydrolytic enzyme production by *F. oxysporum* [57]. The in vitro analysis indicated an inhibition rate of 63 to 98% for cellulase and 91 to 100% for pectinemethyl-esterase. Moreover, phenols from rice straw (mainly coumaric acid and ferulic acid) decreased up to 23% of the cellulase activity of *T. reesei* [58]. Abu-Taleb and his colleagues proved that flavonoid subfractions from *Rumez vesicarius* and *Ziziphus spina-christi* ethanolic extracts (containing rutin, quercitin, apigenin, and kaempherol) inhibit both spore production and cellulase activity of *F. solani* and *Drechslera biseptata* fungal strains [59].

## 4. Materials and Methods

### 4.1. Preparation of Plant Extract

For ultrasound-assisted extraction, a sonication water bath with frequency between 20 and 2000 kHz was used, allowing the cell lysis. Five grams of grounded plant material obtained from Romanian organic agriculture were mixed with 25 mL of ethanol 50%. The obtained extract was filtered and brought to the mark in a 50 mL volumetric flask.

### 4.2. The Total Phenolic Content (TPC) Assay

TPC content was determined by the Folin–Ciocalteu method [60]. Briefly, an aliquot was mixed with 100 µL Folin–Ciocalteu reagent, 900 µL distilled water, and 1000 µL of saturated sodium carbonate. The tubes were vortexed for 15 s and allowed to stand in the dark for 60 min for color development. Absorbance was then measured at 765 nm using a FlexStation 3 UV–VIS (Molecular Devices Company, Sunnyvale, CA, USA) spectrophotometer. A standard curve was prepared by using different concentrations of gallic acid in the same condition with samples (R^2^ = 0.9972). TPC content was expressed as milligram gallic acid equivalent/g plant material (mg GAE/g). Analyses were performed in triplicate.

### 4.3. The Total Flavonoid Content Assay

Flavonoid content was assessed through the AlCl_3_ method [61]. Briefly, 0.1 mL sample/standard solution was mixed with 0.1 mL sodium acetate 10% and 0.12 mL AlCl_3_ 2.5%, with the final volume being adjusted to 1 mL with ethanol 50%. The samples were then vortexed and incubated in the dark for 45 min. The absorbances were measured at λ = 430 nm. A standard curve was prepared by using different concentrations of quercetin (R^2^ = 0.9956). Total flavonoid content was expressed as milligram quercetin equivalent/g dry leaves (mg QE/g DL). Analyses were performed in triplicate.

### 4.4. Total Thiosulfinate Content Assay

A spectrophotometric method based on the reaction of DTNB was used to measure the thiosulfinate content according to [22,62]. An aliquot of 625 μL of 0.8 mM cysteine solution was added to 375 μL of garlic extract or a similar aliquot of distilled water (blank). The samples were kept for 10 min at room temperature, and an aliquot of 200 μL of garlic/cysteine solution or water/cysteine solution was added to 800 μL of 200 μM DTNB, which was prepared in 50 mM HEPES buffer. After shaking, test samples were left for 10 min to allow for color development. Absorbance was measured at 412 nm, and the thiosulfinate concentrations were calculated, taking into account ε = 14,150 M^−1^ × cm ^−1^. Total tiosulfinate content was expressed as µM allicin equivalent/g plant material (mg QA/g). Analyses were performed in triplicate.

### 4.5. Characterization of the Extract by UHPLC–MS/MS

Polyphenolic compound quantification and qualitative identification of thiosulfinate compounds were performed using a high-resolution Q Exactive mass spectrometer™ Focus Hybrid Quadrupole—OrbiTrap (Thermo Fisher Scientific) equipped with HESI, coupled to a high-performance liquid chromatograph UltiMate 3000 UHPLC (ThermoFisher Scientific). Chromatographic separation was performed on a Kinetex^®^ C18 column (100 × 2.1 mm, 1.7 µm particle diameter) at 30 °C. Mobile phase: A—water with 0.1% formic acid and B—methanol with 0.1% formic acid, elution in gradient at a flow rate between 0.3 and 0.4 mL/min. For polyphenolic compound quantification, including phenolic acids, flavonoids, stilbenoids (t-resveratrol), and sesquiterpenoid hormones (abscisic acid), the mass spectra were recorded in negative ionization mode, in a range between *m/z* 100 and 800, at a resolution of 70,000, while for thiosulfinate compounds, the mass spectra were recorded in positive ionization mode. Nitrogen was used as collision gas and auxiliary gas at a flow rate of 11 and 48 arbitrary units, respectively. The applied voltage was 2.5 kV, and the capillary temperature was 320 °C. The energy of the collision cell varied between 30 and 60 eV. For polyphenolic compound quantification, calibration was performed in the concentration range between 0 and 1000 μg/L for each of the phenolic acids and flavonoids by serial dilution with methanol of the standard mixture of concentration 10 mg/L [63]. The data were purchased and processed using the Xcalibur software package (Version 4.1). ChemSpider reference spectral database (www.chemspider.com, accessed on 22 March 2021) was used as a reference library to identify thiosulfinate compounds of interest.

### 4.6. Antioxidant Activity Assay

#### 4.6.1. DPPH Assay

It was performed according to the method of [64] with some slight changes. The reaction mixture consisted of adding 100 µL of sample/standard and 100 µL of 0.3 mM DPPH radical solution in 50% ethanol. The absorbance was measured at λ = 517 nm after 30 min of incubation in the dark using a UV–VIS spectrophotometer. The concentrations used for the Trolox calibration curve were in the range of 5–80 µM (R^2^ = 0.9988).

#### 4.6.2. CUPRAC Method

Copper ion reduction was performed according to a method adapted from [65], as follows: 20 µL of sample/standard solutions were mixed with 60 µL CuSO_4_ (5 mM), neocuproine 60 µL (3.75 mM), and distilled water (560 µL), reaching a final volume of 700 µL. After 30 min, the absorbance was measured at 450 nm. The standard Trolox solutions required for the calibration curve were between 1 and 0 mM (R^2^ = 0.9952).

#### 4.6.3. FRAP Assay

The antioxidant power determination of iron reduction was performed by the method described by Benzie and Strain in 1999 [66], with some modification. The following solutions were prepared: 300 mM acetate buffer (pH 3.6), 10 mM TPTZ stock solution in 40 mM HCl, and 20 mM FeCl_3_ solution in distilled water. The FRAP reagent was obtained by mixing 300 mM acetic acid–sodium acetate buffer (pH 3.6) with 10 mM TPTZ solution and 20 mM FeCl_3_ solution (10:1:1). The FRAP reagent was kept on an incubator at 37 °C until the time of analysis. To 10 µL sample/standard solution, 190 µL FRAP reagent was added, and the mixture was incubated for 30 min at 37 °C. After incubation, the absorbance at λ = 593 nm was read. The calibration curve was performed for the concentration range 0–250 µM Trolox/mL (R^2^ = 0.9978).

### 4.7. Qualitative Screening of the Antimicrobial Activity of the Tested Plant Extract

The qualitative screening of the antimicrobial activity was performed by an adapted diffusion method on Sabouraud agar (SDA) medium inoculated with standard fungal cell suspensions prepared from the following stains: *Penicillium chrysogenum*, *P. corylophylum*, *P. expansum*, *P. digitatum*, *Aspergillus niger*, *A. flavus*, *A. clavatus*, *A. nidulans*, *A. ochraceus*, *A. versicolor*, *A. ustus*, *A. sydowi*, *Rhizopus oryzae*, *T. orientale*, *Byssochlamys spectabilis*, *Alternaria alternata*, *Purpureocillium lilacinum*, *Fusarium proliferatum*, *F. cerealis culmorum* group, and *Cladosporium* spp. The Mueller–Hinton (MH) medium was used to assess the antibacterial activity against the following bacterial strains: *Bacillus pumilus*, *B. megaterium*, *B. cereus*, *B. mojavensis*, *B. atrophaeus*, *B. subtilis*, *B. endophyticus*, *B. thuringiensis*, *A. aurascens, A. globiformis*, *A. bergeri, P. abietaniphila*, *P. koreensis*, and *R. erythropolis* species. The strains were isolated previously from heritage churches, objects, and from two museum collections in Romania during 2018 and 2019 [10]. Subsequently, 10 μL of hydro-alcoholic extract (100 mg/mL), as already presented in Section 2.1, was prepared in sterile water, and solvent control (ethanol 50%) was spotted over it. The plates were incubated for 5–7 days at room temperature for 24 h at 37 °C; then, the growth inhibition diameter zone was measured and the values were converted into arbitrary units using the following convention: 0 value for no inhibition zone, 1 value for a growth inhibition diameter zone up to 10 mm, and 2 value for a growth inhibition diameter zone of 11–20 mm.

### 4.8. Quantitative Evaluation of the Antimicrobial Activity of the Plant Extract

The quantitative evaluation of the antifungal activity was performed in RPMI (Roswell Park Memorial Institute) 1640/MH Broth medium using the microdilution method in 96 multi-well plates [67]. The serial twofold microdilutions of the compounds were achieved in 100 μL of RPMI 1640/MH broth medium seeded with the standard fungal/bacterial inoculum of 0.4–5 × 10^4^ CFU/mL. After incubation for 5–7 days at room temperature for 24 h at 37 °C, the minimum inhibitory concentration (MIC) values were established in correspondence to the lowest concentration, at which the tested plant extract inhibited the growth of the microbial cultures. The final results were calculated and graphically expressed after the solvent (ethanol 50% diluted in the same manner as the extract) inhibitory effect was eliminated.

### 4.9. The Influence of A. sativum Extract on the Microbial Adherence Capacity to the Inert Surface

The influence on the ability of microbial adherence to the inert substratum (96-well plate, untreated polystyrene) was measured after running the quantitative analysis of the antimicrobial activity by evaluating the biofilm biomass, after fixation with methanol and crystal violet staining. The optical density of the biological material resuspended in acetic acid 33% was determined by reading the absorbance at 490 nm. Negative, positive, and solvent controls were used. The positive control highlights the sterile working conditions, where no microbial cell has adhered to the substrate. Positive controls are represented by the natural adhesion of untreated microbial strains. The solvent control concentration was similar with that of ethanol. The experiment was performed in triplicate. The formula used for the evaluation of the microbial adherence capacity was

IIBG% = 100 − (Ae-s × 100)/Ac(1)

where Ae-s = the absorbance of the biofilm formed and treated with extract/solvent, and Ac = the absorbance of the biofilm formed untreated.

### 4.10. The Influence of A. sativum Hydro-Alcoholic Extract on the Production of Microbial Enzymes and Organic Acids Involved in Biodeterioration

Microbial strains treated with subinhibitory concentration of alcoholic extract (MIC/4) were evaluated for their capacity to release different biodegradative enzymes and organic acids. In this purpose, different culture media were spotted with 10 µL suspension adjusted to 1 McFarland prepared from each microbial strain (untreated culture, serving as growth control and treated with A. *sativum*) and then incubated at 26–28 °C for 5–8 days.

Cellulase production was examined on agar plates containing cellulose (CMC, 5 g, Avicel), yeast extract (1 g), Congo red 0.05 g/100 mL, agar 15 g, and distilled water 1000 mL at pH 7.0. A clear zone around the microbial colonies indicates cellulose hydrolysis. Phenoloxidase activity was determined on agar plates containing malt extract (15 g), agar (20 g), 100 mL tannic acid water solution (0.08%) (sterilized by filtration and added after autoclaving), and distilled water up to 1000 mL at pH 7.0 [68]. For the esterase production, agar plates containing peptone (20 g), NaCl (10 g), CaCl (0.2 g), Tween 20 (20 mL) (added after sterilization), agar (30 g), and distilled water 1000 mL at pH 5.4 [69] were used. For the acid production, agar plates containing sodium nitrate (2 g), dipotassium phosphate (1 g), magnesium sulphate (0.5 g), potassium chloride (0.5 g), ferrous sulphate (0.01 g), glucose (10 g), phenol red (0.003 g), agar (20 g), and distilled water 1000 mL at pH 7.0 [70] were used.

The inhibitory activity was semi-quantitatively evaluated by measuring the ratio of the colony diameter (C) to the diameter of the specific inhibition zone occurring around the colony (D) (e.g., clear zone for cellulase activity, a precipitation zone for phenoloxidase, a brown zone for esterase, and a change of color from red to yellow in the area in the immediate vicinity of the culture spot for organic acids).

Finally, the influence of *A. sativum* extract and solvent control (the same ethanol concentration as the extract used) on cellulose, esterase, phenoloxidase, and organic acid production was quantified by the following equation:

$$\mathrm{Inhibition}\;(\%)=\frac{D2-C2}{D1-C1}\times 100,$$

where *C*1—colony diameter of control strain, *D*1—inhibitory effect zone diameter of strain control, *C*2—colony diameter of treated strain, and *D*2—inhibitory effect zone diameter of treated strain.

### 4.11. Statistical Analysis

Data were expressed as means ± SD determined by triplicate analysis. The statistical analysis was conducted using GraphPad Prism 9. Data were analyzed using unpaired *t*-test with Welch correction of multiple comparisons for antibiofilm activity and enzymatic inhibition activity, and no correction for multiple comparisons in the case of antimicrobial activity. The level of significance was set to *p* < 0.05.

## 5. Conclusions

To the best of our knowledge, this is the first study conducted to demonstrate the efficiency of the garlic extract against a significant number of fungal and bacterial biodeteriogenic strains isolated from different surfaces of cultural heritage objects and buildings from outdoor and indoor environments and to assess its inhibitory activity not only on microbial growth, but also on biofilm development and production of different factors involved in biodeterioration. The study design also allowed us to stratify the efficiency of the garlic extract depending on the genera and species, but also on the type of material/surface. The main compounds identified in the garlic extract were thiosulfinate, flavonoids, and polyphenols. The most susceptible species were *B. spectabilis*, *Cladosporium* spp., and *T. orientale.* From the species of the most frequently isolated genera *(Penicillium* spp. and *Aspergillus* spp.)*,* the *A. sativum* plant extract demonstrated the highest efficiency against the *A. versicolor*, *A. ochraceus*, *P. expansum*, and *A. niger* strains. Fungi isolated from paintings and paper as well as bacteria from wood, paper, and textiles responded better to the garlic extract treatments, as revealed by the low MIC values. The tested extract inhibited the adherence capacity and the production of biodeteriogenic products, such as cellulase, organic acids, and esterase. The results of this study show the great potential of the tested extract on the conservation and, thus, on the safeguarding of monuments by maintaining the carpentry and masonry in a stage that allows for the transmission of this tangible heritage to future generations.

## Figures and Tables

**Table 1:** The total polyphenol content (TPC) and antioxidant activity obtained by different methods.

Parameter	The Analyzed Extract in Relation to the Quantity Used (Mean ± SD)
TPC (mg GAE/g)	0.95 ± 0.011
Flavonoids (μg quercetin/g)	64.33 ± 7.69
Total thiosulfinate (µM/g)	307.66 ± 0.043
Antioxidant activity	Extract (0.1 g/mL)
DPPH (μM Trolox/mL)	66.39 ± 5.50
CUPRAC (μM Trolox/mL)	52.25 ± 3.01
FRAP (μM Trolox/mL)	43.51 ± 1.49

**Table 2:** Identification and quantitative data regarding the bioactive compounds in the *A. sativum* liquid extract.

No.	Compound	Retention Time (min)	Accurate Mass [M − H]^−^/[M − H]^+^	Mass Fragments	Concentration (µg/L)
**Polyphenolic compounds by UHPLC-MS/MS in negative ionization mode**					
1	Gallic acid	0.68	169.0133	125.0231	0.4
2	3,4-Dihydroxybenzoic acid	1.59	153.0183	109.0281	0.8
3	4-Hydroxybenzoic acid	5.40	137.0232	93.0331	4
4	Chlorogenic acid	7.55	353.0879	191.0553	0.8
5	Syringic acid	8.03	197.0450	182.0212, 166.9976, 153.0547, 138.0311, 123.0075	7.2
6	Caffeic acid	8.08	179.0338	135.044	6.4
7	Vanillic acid	8.31	167.0343	152.0105, 124.0154, 111.0075, 139.0025, 95.0125	65.2
8	*p*-Coumaric acid	8.59	163.0392	119.0489	1.2
9	*t*-Ferulic acid	8.83	193.0500	178.0262, 134.0361	18.8
10	Ellagic acid	9.66	300.9990	300.9990	13.2
11	Cinnamic acid	10.45	147.0441	119.0489, 103.0387	7.6
12	Abscisic acid	10.04	263.1288	179.9803, 191.9454	2.4
**Ʃ phenolic acids**					**128**
13	Epi-catechin	7.98	289.0719	109.0282, 125.0232, 137.0232, 151.0390, 203.0708, 245.0817	41.2
14	Quercetin	10.74	301.0356	151.0226, 178.9977, 121.0282, 107.0125	0.8
15	Naringin	9.25	579.1718	363.0721	3.6
16	Hesperidin	9.37	609.1824	377.0876	14
17	Rutin	9.43	609.1462	3345.0614	10
18	Kaempferol	11.62	285.0406	151.0389, 117.0180	2.8
19	Isorhamnetin	11.80	315.0512	300.0276	9.6
20	Apigenin	11.86	269.0457	117.0333, 151.0027, 107.0126	4.4
21	Pinocembrin	12.70	255.0663	213.0551, 151.0026, 107.0125	1.6
22	Chrysin	13.52	253.0506	143.0491, 145.0284, 107.0125, 209.0603, 63.0226, 65.0019	3.6
23	Galangin	13.77	269.0458	169.0650, 143.0491	2.8
24	Pinostrobin	14.84	269.081	179.0554	2.8
**Ʃ flavonoids**					**97.2**
25	*t*-Resveratrol	9.55	227.0707	185.0813, 143.0337	0.8
**Thiosulfinate compounds by UHPLC-MS/MS in positive ionization mode**					
1	Alliin	0.68	178.0530	175.1188, 116.0709	-
2	Gamma-glutamyl-S-methylcysteine	1.05	265.0850	182.0811, 132.1020	-
3	Gamma-glutamyl (S)-allylcysteine	3.61	291.1005	162.0582, 139.0502	-
4	Gamma-glutamyl-S-trans-propenyl cysteine	5.68	291.1006	162.0582	-
5	Allicin	8.19	163.0245	120.9779	-

**Table 3:** The MIC and MBEC values of the *A. sativum* hydro-alcoholic extract.

Species	Isolation Source	MIC (mg/mL)	MBEC ^1^ (mg/mL)	IIBG ^2^ (%)	*p* Value (Relative to Solvent Control)
*A. niger*	textiles	25	25	95.08 ± 0.91	<0.0001
*P. chrysogenum*	textiles	25	6.25	89.11 ± 0.91	<0.01
*Penicilium* spp.	stone	25	12.5	90.71 ± 0.70	<0.001
*P. corylophilum*	textiles	25	6.25	80.47 ± 2.73	<0.05
*A. flavus*	textiles	25	12.5	88.57 ± 1.30	<0.01
*Aspergillus* spp.	stone	25	6.25	90.26 ± 1.01	<0.000001
*A. sydowii*	stone	50	12.5	93.54 ± 1.09	<0.01
*A. niger*	wood	6.25	12.5	88.74 ± 1.31	<0.001
*A. flavus*	textiles	12.5	6.25	88.19 ± 2.40	<0.01
*P. lilacium*	stone	12.5	NA		
*P. lilacium*	stone	12.5	NA		
*Aspergillus* spp.	stone	25	25	84.28 ± 1.86	<0.01
*P. chrysogenum*	wood	50	12.5	93.87 ± 0.03	0.072
*P. corylophilum*	wood	12.5	6.25	92.50 ± 0.00	<0.00001
*P. corylophilum*	wood	6.25	12.5	92.30 ± 1.92	<0.0001
*P. corylophilum*	wood	25	12.5	93.42 ± 1.38	<0.001
*A. niger*	textiles	25	12.5	91.96 ± 0.71	<0.001
*B. megaterium*	wood	6.25	NA		
*B. subtilis*	wood	6.25	NA		
*B. cereus*	ceramic	1.56	NA		
*B. pumilus*	wood	0.78	NA		
*P. koreensis*	stone	12.5	NA		
*B. arthopahaeus*	stone	6.25	12.5	44.62 ± 7.30	0.342
*B. megaterium*	wood	3.125	NA		
*B. thuringiensis*	stone	3.125	12.5	87.56 ± 4.60	<0.01
*B. pumilus*	stone	6.25	NA		
*A. aurascens*	wood	12.5	NA		
*B. cereus*	wood	3.125	NA		
*B. megaterium*	wood	3.125	NA		
*B. megaterium*	wood	3.125	NA		
*B. pumilus*	painting	12.5	NA		
*R. erythrophilus*	wood	3.125	NA		
*B. pumilus*	wood	3.125	NA		
*B. megaterium*	textiles	6.25	NA		
*A. globiformis*	wood	6.25	NA		
*B. cereus*	wood	6.25	12.5	57.72 ± 5.11	<0.05

^1^ MBEC—minimum biofilm eradication concentration on inert substrate; ^2^ IIBG—inhibition index of biofilm growth; NA—non-adherent.

**Table 4:** The influence of *A. sativum* extract and ethanol (50%) control on the production of enzymatic factors involved in biodeterioration.

**Fungal Strains**										
**Species**	**Isolation Sources**	**Cellulase**			**Esterase**			**Acid Production**		
		***A. sativum* Extract**	**Ethanol 50%**	***p*-Value**	***A. sativum* Extract**	**Ethanol 50%**	***p*** **-Value**	***A. Sativum* Extract**	**Ethanol 50%**	***p*** **-Value**
*A. niger*	textile	105.00 ± 8.66	140.00 ± 17.32	0.0535	66.67 ± 4.44	58.97 ± 4.44	0.1012	79.09 ± 3.86	92.73 ± 0.00	<0.05
*P. chrysogenum*	textile	111.77 ± 5.09	108.82 ± 5.09	0.5185	-	-	-	70.37 ± 8.49	98.15 ± 3.21	<0.05
*Penicilium* spp.	stone	272.73 ± 0.01	327.27 ± 0.05	<0.0001	78.26 ± 13.04	86.96 ± 19.92	0.5666	116.67 ± 5.77	96.67 ± 11.55	0.0765
*P. corylophilum*	textile	105.56 ± 7.86	107.41 ± 6.42	0.7683	-	-	-	-	-	-
*A. flavus*	textile	-	-	-	108.00 ± 0.00	88.00 ± 6.93	<0.05	83.91 ± 7.18	81.61 ± 3.98	0.6596
*Aspergillus* spp.	stone	108.16 ± 3.53	106.16 ± 12.75	0.8113	-	-	-	72.00 ± 12.00	104 ± 18.33	0.0746
*A. sydowii*	stone	143.75 ± 8.84	150.00 ± 21.65	0.1918	-	-	-	68.18 ± 6.43	60.61 ± 5.24	0.6788
*A. niger*	wood	-	-	-	-	-	-	92.00 ± 13.86	136.00 ± 6.93	<0.05
*A. flavus*	textile	87.50 ± 8.66	90.00 ± 12.99	0.7972	97.96 ± 6.12	79.59 ± 0.00	<0.05	63.16 ± 0.00	68.42 ± 4.56	0.1835
*P. lilacium*	stone	76.92 ± 0.00	100.00 ± 10.88	0.0667	94.44 ± 9.62	111.11 ± 9.62	0.1012	110.71 ± 6.19	103.57 ± 6.19	0.2302
*P. lilacium*	stone	118.52 ± 16.97	133.33 ± 0.00	0.4226	93.75 ± 0.00	87.50 ± 10.83	0.2697	137.5 ± 5.41	118.75 ± 5.41	<0.05
*Aspergillus* spp.	stone	97.50 ± 13.00	60.00 ± 7.50	<0.05	94.29 ± 0.00	62.86 ± 4.95	<0.01	76.67 ± 5.77	130 ± 17.32	<0.05
*P. chrysogenum*	wood	93.94 ± 6.31	93.94 ± 9.26	>1	-	-	-	82.14 ± 6.19	60.71 ± 6.19	<0.05
*P. corylophilum*	wood	126.67 ± 11.55	70.00 ± 14.14	<0.01	-	-	-	144.44 ± 19.25	120.83 ± 5.89	0.1589
*P. corylophilum*	wood	71.80 ± 11.75	97.44 ± 8.88	<0.05	-	-	-	-	-	-
*A. niger*	textile	-	-	-	-	-	-	138.46 ± 0.01	115.39 ± 0.11	<0.0001
**Bacterial Strains**										
**Species**	**Isolation Sources**	**Cellulase**			**Esterase**			**Acid Production**		
		***A. sativum* Extract**	**Ethanol 50%**	***p*-Value**	***A. sativum* Extract**	**Ethanol 50%**	***p*-Value**	***A. sativum* Extract**	**Ethanol 50%**	***p*-Value**
*B. megaterium*	wood	-	-	-	120.00 ± 0.00	90.00 ± 42.43	0.3454	100.00 ± 11.47	105.00 ± 7.50	0.5667
*B. subtilis*	wood	-	-	-	100.00 ± 21.65	0	<0.05	-	-	-
*P. koreensis*	stone	-	-	-	-	-	-	89.80 ± 3.53	95.92 ± 3.53	0.1013
*B. arthopahaeuss*	stone	-	-	-	71.43 ± 12.37	78.57 ± 12.37	0.5186	-	-	-
*B. megaterium*	wood	-	-	-	-	-	-	183.33 ± 23.57	183.33 ± 23.57	>1
*B. cereus*	wood	-	-	-	75.00 ± 9.64	68.19 ± 0.00	0.3457	-	-	-
*B. pumilus*	painting	-	-	-	116.67 ± 57.70	0	0.0728	93.333 ± 11.55	106.67 ± 11.55	0.2301
*B. megaterium*	textile	-	-	-	-	-	-	0	75.76 ± 13.89	<0.05
*B. cereus*	wooden	-	-	-	87.50 ± 5.41	75.00 ± 0.00		0	76.67 ± 4.71	<0.01

## Supplementary Materials

The following are available online. Figure S1. *Allium sativum* inhibitory activity against fungi and bacteria (in percent) expression by arbitrary units of inhibition zone diameter. Figure S2. Allium sativum inhibitory activity against fungi and bacteria (in percent) expressed by arbitrary units of growth inhibition zones diameter.

## Sample Availability

Samples of the compounds are available from the authors.

## References

[1] Rădulescu H.C., Perdum E., Mitran C.E., Dincă L.C., Lazăr V. (2018). Biodeterioration capacity of a microfungal species isolated from textile cultural heritage items on contemporary wool materials. Publ. House Rom. Acad. Ser. B.

[2] Sterflinger K. (2010). Fungi: Their role in deterioration of cultural heritage. Fungal Biol. Rev..

[3] Savković Ž., Stupar M., Unković N., Knežević A., Vukojević J., Grbić M.L. (2021). Fungal Deterioration of Cultural Heritage Objects. Biodegradation.

[4] Moza M.I., Mironescu M., Georgescu C., Florea A., Bucşa L. (2012). Isolation and characterisation of moulds degrading mural paintings. Ann. Rscb.

[5] Lupan I., Ianc M., Kelemen B., Carpa R., Rosca-Casian O., Chiriac M., Popescu O. (2014). New and old microbial communities colonizing a seventeenth-century wooden church. Folia Microbiol..

[6] IIies D., Onet A., Wendt J.A., Ilieş M., Timar A., Ilies A., Baias Ş., Herman G. (2018). Study on microbial and fungal contamination of air and wooden surfaces inside of a historical Church from Romania. J. Environ. Biol..

[7] Rădulescu H.C., Gheorghe I., Gradisteanu G., Ispas A., Popescu C., Roşu G., Chifiriuc M.C., Lazăr V. (2019). Molecular characterization based on Internal Transcribed Spacer (ITS) marker sequence of fungal strains isolated from heritage ethnographic textiles. Rom. Biotechnol. Lett..

[8] Ilieș D.C., Oneț A., Grigore H., Liliana I., Alexandru I., Ligia B., Ovidiu G., Florin M., Ștefan B., Tudor C. (2019). Exploring the indoor environment of heritage buildings and its role in the conservation of valuable objects. Environ. Eng. Manag. J. EEMJ.

[9] Sirghi A.C., Gheorghe I., Sarbu I., Marutescu L., Stoian G., Zhiyong Z., Chifiriuc M.C. (2018). Identification of fungal strains isolated from buildings of cultural importance in Romania and antagonistic relationships amongst them. Rom. Biotechnol. Lett..

[10] Gheorghe I., Sârbu I., Pecete I., Blăjan I., Balotescu I. (2020). Multi-level characterization of microbial consortia involved in the biodeterioration of wooden and stone romanian heritage churches. Conserv. Sci. Cult. Herit..

[11] Palla F., Bruno M., Mercurio F., Tantillo A., Rotolo V. (2020). Essential oils as natural biocides in conservation of cultural heritage. Molecules.

[12] Rosado T., Silva M., Dias L., Candeias A., Gil M., Mirão J., Pestana J., Caldeira A.T. (2017). Microorganisms and the integrated conservation-intervention process of the renaissance mural paintings from Casas Pintadas in Évora–Know to act, act to preserve. J. King Saud Univ. Sci..

[13] Gheorghe I., Avram I., Matis Corbu V., Măruţescu L., Popa M., Balotescu I., Blăjan I., Mateescu V., Zaharia D., Dumbravă A.Ş. (2021). In vitro evaluation of MgB2 powders as novel tools to fight fungal biodeterioration of heritage buildings and objects. Front. Mater..

[14] Yetgin A., Canlı K., Altuner E.M. (2018). Comparison of antimicrobial activity of *Allium sativum* cloves from China and Taşköprü, Turkey. Adv. Pharmacol. Sci..

[15] Grumezescu A.M., Andronescu E., Holban A.M., Ficai A., Ficai D., Voicu G., Grumezescu V., Balaure P.C., Chifiriuc C.M. (2013). Water dispersible cross-linked magnetic chitosan beads for increasing the antimicrobial efficiency of aminoglycoside antibiotics. Int. J. Pharm..

[16] Fierascu I., Dima R., Fierascu R.C. Natural Extracts for preventing Artefacts Biodeterioration. Proceedings of the International Conference on Cultural Heritage and New Technologies Vienna.

[17] Fierascu I., Ion R.M., Radu M., Bunghez I., Avramescu S., Fierascu R. (2014). Comparative study of antifungal effect of natural extracts and essential oils of *Ocimum basilicum* on selected artefacts. Rev. Roum. Chim..

[18] Wu X., Santos R.R., Fink-Gremmels J. (2015). Analyzing the antibacterial effects of food ingredients: Model experiments with allicin and garlic extracts on biofilm formation and viability of *Staphylococcus epidermidis*. Food Sci. Nutr..

[19] Woods-Panzaru S., Nelson D., McCollum G., Ballard L.M., Millar B.C., Maeda Y., Goldsmith C.E., Rooney P.J., Loughrey A., Rao J.R. (2009). An examination of antibacterial and antifungal properties of constituents described in traditional Ulster cures and remedies. Ulst. Med. J..

[20] Khan S., Imran M., Imran M., Pindari N. (2017). Antimicrobial activity of various ethanolic plant extracts against pathogenic multi drug resistant *Candida* spp.. Bioinformation.

[21] Santos D.I., Neiva Correia M.J., Mateus M.M., Saraiva J.A., Vicente A.A., Moldão M. (2019). Fourier transform infrared (FT-IR) spectroscopy as a possible rapid tool to evaluate abiotic stress effects on pineapple by-products. Appl. Sci..

[22] Gonzalez R.E., Soto V.C., Sance M.M., Camargo A.B., Galmarini C.R. (2009). Variability of solids, organosulfur compounds, pungency and health-enhancing traits in garlic (*Allium sativum* L.) cultivars belonging to different ecophysiological groups. J. Agric. Food Chem..

[23] Martínez G., Regente M., Jacobi S., Del Rio M., Pinedo M., de la Canal L. (2017). Chlorogenic acid is a fungicide active against phytopathogenic fungi. Pestic. Biochem. Physiol..

[24] Zhu C., Lei M., Andargie M., Zeng J., Li J. (2019). Antifungal activity and mechanism of action of tannic acid against *Penicillium digitatum*. Physiol. Mol. Plant Pathol..

[25] Miron T., Rabinkov A., Mirelman D., Wilchek M., Weiner L. (2000). The mode of action of allicin: Its ready permeability through phospholipid membranes may contribute to its biological activity. Biochim. Biophys. BBA Biomembr..

[26] Gruhlke M.C., Hemmis B., Noll U., Wagner R., Lühring H., Slusarenko A.J. (2015). The defense substance allicin from garlic permeabilizes membranes of Beta vulgaris, Rhoeo discolor, Chara corallina and artificial lipid bilayers. Biochim. Biophys. Acta BBA Gen. Subj..

[27] Leontiev R., Hohaus N., Jacob C., Gruhlke M.C., Slusarenko A.J. (2018). A comparison of the antibacterial and antifungal activities of thiosulfinate analogues of allicin. Sci. Rep..

[28] Negri M., Salci T.P., Shinobu-Mesquita C.S., Capoci I.R., Svidzinski T.I., Kioshima E.S. (2014). Early state research on antifungal natural products. Molecules.

[29] Choo S., Chong P., Tay S., Wong E., Madhavan P., Yong P. (2020). Inhibition of sessile and biofilm growth in various *Aspergillus* species by allicin associated with disruption to structural changes in cell wall. Int. J. Infect. Dis..

[30] Kim Y.-S., Kim K.S., Han I., Kim M.-H., Jung M.H., Park H.-K. (2012). Quantitative and qualitative analysis of the antifungal activity of allicin alone and in combination with antifungal drugs. PLoS ONE.

[31] Gruhlke M.C., Portz D., Stitz M., Anwar A., Schneider T., Jacob C., Schlaich N.L., Slusarenko A.J. (2010). Allicin disrupts the cell’s electrochemical potential and induces apoptosis in yeast. Free Radic. Biol. Med..

[32] Aala F., Yusuf U.K., Nulit R., Rezaie S. (2014). Inhibitory effect of allicin and garlic extracts on growth of cultured hyphae. Iran. J. Basic Med. Sci..

[33] Salehi B., Zucca P., Orhan I.E., Azzini E., Adetunji C.O., Mohammed S.A., Banerjee S.K., Sharopov F., Rigano D., Sharifi-Rad J. (2019). Allicin and health: A comprehensive review. Trends Food Sci. Technol..

[34] Rahman M.S. (2007). Allicin and other functional active components in garlic: Health benefits and bioavailability. Int. J. Food Prop..

[35] Sarfraz M., Nasim M.J., Jacob C., Gruhlke M.C. (2020). Efficacy of allicin against plant pathogenic fungi and unveiling the underlying mode of action employing yeast based chemogenetic profiling approach. Appl. Sci..

[36] Curtis H., Noll U., Störmann J., Slusarenko A.J. (2004). Broad-spectrum activity of the volatile phytoanticipin allicin in extracts of garlic (*Allium sativum* L.) against plant pathogenic bacteria, fungi and Oomycetes. Physiol. Mol. Plant Pathol..

[37] Getti G., Poole P. (2019). Allicin causes fragmentation of the peptidoglycan coat in Staphylococcus aureus by effecting synthesis and aiding hydrolysis: A determination by MALDI-TOF mass spectrometry on whole cells. J. Med. Microbiol..

[38] Mattio L., Catinella G., Iriti M., Vallone L. (2021). Inhibitory activity of stilbenes against filamentous fungi. Ital. J. Food Saf..

[39] Oliveira V., Carraro E., Auler M.E., Khalil N.M. (2016). Quercetin and rutin as potential agents antifungal against *Cryptococcus* spp.. Braz. J. Biol..

[40] Lee W., Woo E.R., Lee D.G. (2016). Phytol has antibacterial property by inducing oxidative stress response in *Pseudomonas aeruginosa*. Free Radic. Res..

[41] Pei K., Ou J., Huang J., Ou S. (2016). p-Coumaric acid and its conjugates: Dietary sources, pharmacokinetic properties and biological activities. J. Sci. Food Agric..

[42] Zabka M., Pavela R. (2013). Antifungal efficacy of some natural phenolic compounds against significant pathogenic and toxinogenic filamentous fungi. Chemosphere.

[43] Palla F., Rotolo V., Giordano A. (2019). Biotechnology a Source of Knowledge in Agreement with Green Strategies for the Conservation of Cultural Assets. Conserv. Sci. Cult. Herit..

[44] Kutawa A.B., Danladi M.D., Haruna A. (2018). Regular article antifungal activity of garlic (*Allium sativum*) extract on some selected fungi. J. Med. Herbs Ethnomed..

[45] Rotolo V., Barresi G., Di Carlo E., Giordano A., Lombardo G., Crimi E., Costa E., Bruno M., Palla F. (2016). Plant extracts as green potential strategies to control the biodeterioration of cultural heritage. Int. J. Conserv. Sci..

[46] Dakal T.C., Cameotra S.S. (2012). Microbially induced deterioration of architectural heritages: Routes and mechanisms involved. Environ. Sci. Eur..

[47] Deepika M.S., Thangam R., Sakthidhasan P., Arun S., Sivasubramanian S., Thirumurugan R. (2018). Combined effect of a natural flavonoid rutin from Citrus sinensis and conventional antibiotic gentamicin on *Pseudomonas aeruginosa* biofilm formation. Food Control.

[48] Shao D., Li J., Li J., Tang R., Liu L., Shi J., Huang Q., Yang H. (2015). Inhibition of gallic acid on the growth and biofilm formation of *Escherichia coli* and *Streptococcus mutans*. J. Food Sci..

[49] Teodoro G.R., Gontijo A.V., Salvador M.J., Tanaka M.H., Brighenti F.L., Delbem A.C., Delbem Á.C., Koga-Ito C.Y. (2018). Effects of acetone fraction from *Buchenavia tomentosa* aqueous extract and gallic acid on *Candida albicans* biofilms and virulence factors. Front. Microbiol..

[50] Myszka K., Schmidt M.T., Białas W., Olkowicz M., Leja K., Czaczyk K. (2016). Role of gallic and p-coumaric acids in the AHL-dependent expression of flgA gene and in the process of biofilm formation in food-associated *Pseudomonas fluorescens* KM120. J. Sci. Food Agric..

[51] Cruz-Villalón G., Pérez-Giraldo C. (2011). Effect of allicin on the production of polysaccharide intercellular adhesin in *Staphylococcus epidermidis*. J. Appl. Microbiol..

[52] Lihua L., Jianhui W., Jialin Y., Yayin L., Guanxin L. (2013). Effects of allicin on the formation of *Pseudomonas aeruginosa* biofilm and the production of quorum-sensing controlled virulence factors. Pol. J. Microbiol..

[53] Girish V.M., Liang H., Aguilan J.T., Nosanchuk J.D., Friedman J.M., Nacharaju P. (2019). Anti-biofilm activity of garlic extract loaded nanoparticles. Nanomed. Nanotechnol. Biol. Med..

[54] Mazzoli R., Giuffrida M.G., Pessione E. (2018). Back to the past: “Find the guilty bug—Microorganisms involved in the biodeterioration of archeological and historical artifacts”. Appl. Microbiol. Biotechnol..

[55] Cappitelli F., Pasquariello G., Tarsitani G., Sorlini C. (2010). Scripta manent? Assessing microbial risk to paper heritage. Trends Microbiol..

[56] Singh A.P. (2012). A review of microbial decay types found in wooden objects of cultural heritage recovered from buried and waterlogged environments. J. Cult. Herit..

[57] El Hassni M., Laadouzaa H., El Hadrami A., Dihazi A., Rakibi Y., Lemjiber N., Naamani K. (2021). An in vitro evaluation of the effect of hydroxycinnamic acids on the growth and hydrolytic enzyme production in *Fusarium oxysporum* f. sp. *albedinis*. Arch. Phytopathol. Plant Prot..

[58] Zheng W., Zheng Q., Xue Y., Hu J., Gao M.-T. (2017). Influence of rice straw polyphenols on cellulase production by *Trichoderma reesei*. J. Biosci. Bioeng..

[59] Abu-Taleb A.M., El-Deeb K., Al-Otibi F.O. (2011). Assessment of antifungal activity of *Rumex vesicarius* L. and *Ziziphus spina-christi* (L.) Willd. extracts against two phytopathogenic fungi. Afr. J. Microbiol. Res..

[60] Singleton V.L., Orthofer R., Lamuela-Raventós R.M. (1999). Analysis of total phenols and other oxidation substrates and antioxidants by means of folin-ciocalteu reagent. Methods Enzymol..

[61] Formagio A.S., Kassuya C.A., Neto F.F., Volobuff C.R., Iriguchi E.K., do C Vieira M., Foglio M.A. (2013). The flavonoid content and antiproliferative, hypoglycaemic, anti-inflammatory and free radical scavenging activities of *Annona dioica* St. Hill. BMC Complement. Altern. Med..

[62] Mansor N., Herng H.J., Samsudin S.J., Sufian S., Uemura Y. (2016). Quantification and characterization of allicin in garlic extract. J. Med. Bioeng..

[63] Marinas I.C., Oprea E., Geana E.-I., Tutunaru O., Pircalabioru G.G., Zgura I., Chifiriuc M.C. (2021). Valorization of *Gleditsia triacanthos* Invasive Plant Cellulose Microfibers and Phenolic Compounds for Obtaining Multi-Functional Wound Dressings with Antimicrobial and Antioxidant Properties. Int. J. Mol. Sci..

[64] Madhu G., Bose V.C., Aiswaryaraj A., Maniammal K., Biju V. (2013). Defect dependent antioxidant activity of nanostructured nickel oxide synthesized through a novel chemical method. Colloids Surf. A Physicochem. Eng. Asp..

[65] Meng J., Fang Y., Zhang A., Chen S., Xu T., Ren Z., Han G., Liu J., Li H., Zhang Z. (2011). Phenolic content and antioxidant capacity of Chinese raisins produced in Xinjiang Province. Food Res. Int..

[66] Benzie I.F., Strain J.J. (1996). The ferric reducing ability of plasma (FRAP) as a measure of “antioxidant power”: The FRAP assay. Anal. Biochem..

[67] Najee H., Kamerzan C., Marutescu L., Gheorghe I., Popa M., Gradisteanu G., Lazar V. (2018). Antifungal activity of some medicinal plant extracts against Candida albicans nosocomial isolates. Rom. Biotechnol. Lett..

[68] Muhsin T.M., Al-Zubaidy S.R., Ali E.T. (2001). Effect of garlic bulb extract on the growth and enzymatic activities of rhizosphere and rhizoplane fungi. Mycopathologia.

[69] Zajc J., Gostinčar C., Černoša A., Gunde-Cimerman N. (2019). Stress-tolerant yeasts: Opportunistic pathogenicity versus biocontrol potential. Genes.

[70] Borrego S., Molina A., Santana A. (2017). Fungi in archive repositories environments and the deterioration of the graphics documents. EC Microbiol..

