# Supplementary material for: Demonstration of Allium sativum Extract Inhibitory Effect on Biodeteriogenic Microbial Strain Growth, Biofilm Development, and Enzymatic and Organic Acid Production
Source: Molecules. 2021 Nov 27;26(23):7195. doi: 10.3390/molecules26237195 (PMC8659052; doi:10.3390/molecules26237195)
Supplement: Supplementary file 1 [file molecules-26-07195-s001.zip › molecules-1464404-supplementary.pdf]

molecules

MDPI

Supplementary material submitted to

Special Issue "Extraction, Characterization, and Potential Applications of Bioactive Molecules from Natural Sources II"

Demonstration of Allium sativum Extract Inhibitory Effect on Biodeteriogenic Microbial Strain Growth, Biofilm Development, and Enzymatic and Organic Acid Production

Viorica Maria Corbu [1,2,3,†], Irina Gheorghe [2,4,*,†], Ioana Cristina Marinaș [2,*], Elisabeta Irina Geană [5], Maria Iasmina Moza [2,3,4], Ortansa Csutak [1,3] and Mariana Carmen Chifiriuc [2,4,6,7]

[1] Department of Genetics, Faculty of Biology, University of Bucharest, Botanical Garden, 3 Intrarea Portocalelor St., Bucharest, Romania; viorica.corbu@yahoo.com (V.M.C.); cs.ortansa@gmail.com (O.C.)
[2] Research Institute of the University of Bucharest—ICUB, 91-95 Splaiul Independenței St., District 5, 050095 Bucharest, Romania; iasmina_moza@yahoo.com (M.I.M.); carmen.chifiriuc@gmail.com (M.C.C.)
[3] Doctoral School of Biology, University of Bucharest, 91-95 Splaiul Independenței St., District 5, 050095 Bucharest, Romania
[4] Department of Microbiology and Immunology, Faculty of Biology, University of Bucharest, Botanical Garden,
3 Intrarea Portocalelor St., District 6, 060101, Bucharest, Romania
[5] National R&D Institute for Cryogenics and Isotopic Technologies—ICIT, Rm. Valcea, 4 Uzinei St., PO Raureni Box 7, 240050 Ramnicu Valcea, Romania; irina.geana@icsi.ro
[6] Romanian Academy of Scientists, 54 Spl. Independentei St., District 5, 50085 Bucharest, Romania
[7] The Romanian Academy, 25, Calea Victoriei, sector 1, District1, 010071, Bucharest, Romania
* Correspondence: Correspondence: irina.gheorghe@bio.unibuc.ro (I.G.); ioana.cristina.marinas@gmail.com (I.C.M.)
† These authors have contributed equally to this work.

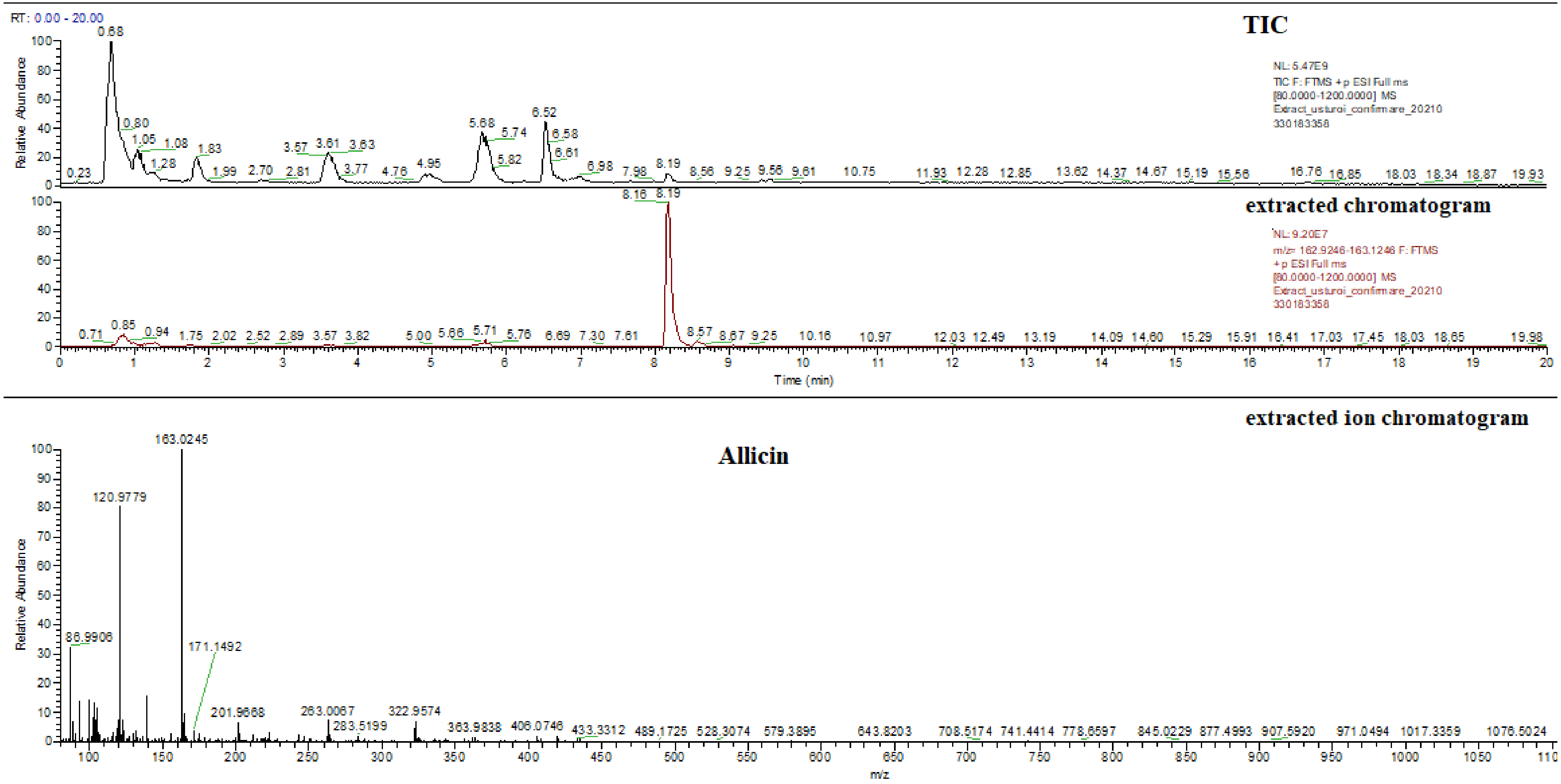

Figure S1. TIC, the extracted chromatogram of the allicin from *A. sativum* plant extract (the chromatogram was extracted from TIC using a 5 ppm mass accuracy window; positive ion mode, full scan, base peak in the range 80-1200 m/z) and the extracted ion chromatogram for m/z 163.0245

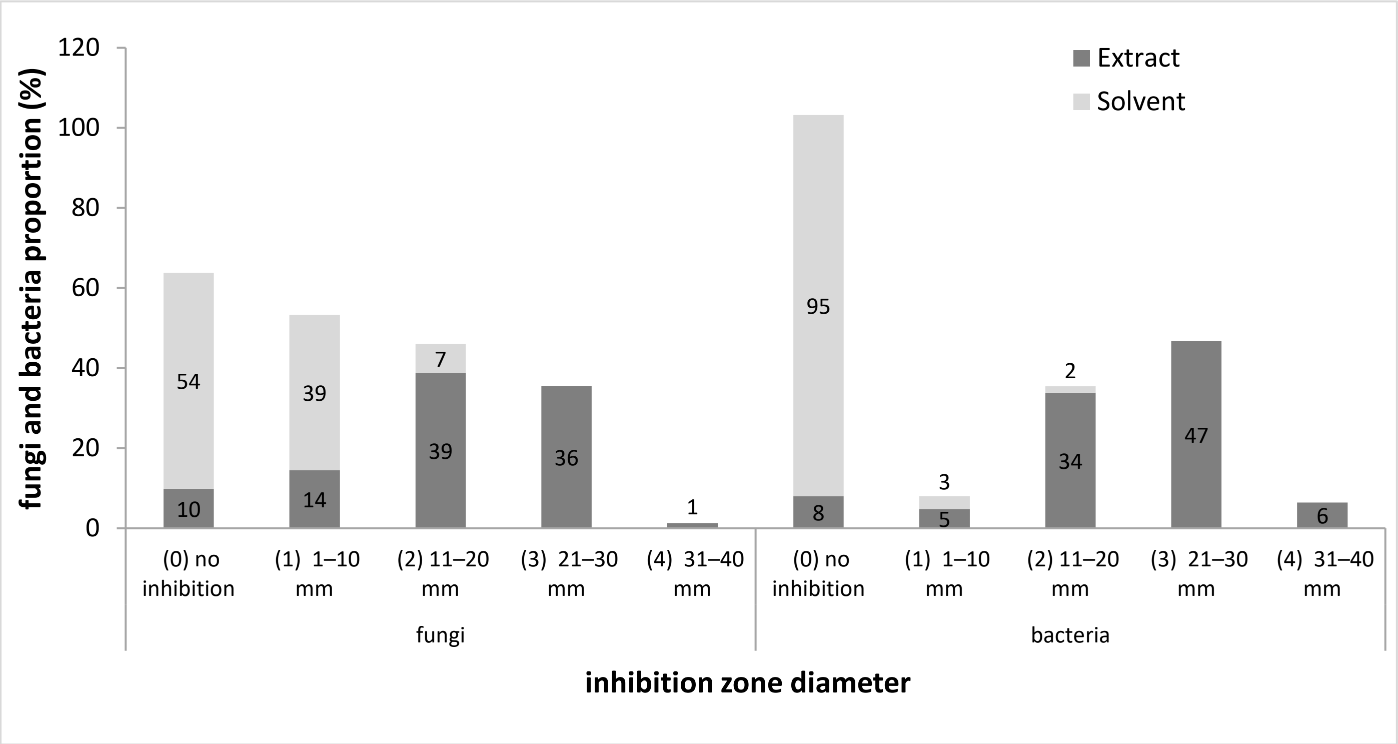

Figure S2. *Allium sativum* inhibitory activity against fungi and bacteria (in percent) expressed by arbitrary units of growth inhibition zones diameter.
